# Education Research: A Qualitative Analysis of Communication-Focused Feedback Provided to Child Neurology Residents During an Objective Structured Clinical Examination

**DOI:** 10.1212/NE9.0000000000200187

**Published:** 2025-02-10

**Authors:** Cindy Ho, Pedro Weisleder, Margie A. Ream, Dara V.F. Albert

**Affiliations:** From the Division of Child Neurology, Department of Pediatrics, Nationwide Children's Hospital/The Ohio State University, Columbus.

## Abstract

**Background and Objectives:**

Child neurology is a specialty with unique challenges in communication. Child neurologists face many complex disorders with a wide array of prognoses and treatments as well as the need to communicate at various developmental levels. Limited literature exists regarding effective communication training during child neurology residency. Our aim was to evaluate feedback provided to child neurology residents by standardized patients (SPs) and faculty during a communication-focused objective structured clinical examination (OSCE) for common themes and identify which elements of communication feedback are most valuable to the residents.

**Methods:**

The child neurology residency at Nationwide Children's Hospital previously developed a set of OSCE cases to assess residents' communication skills. Using a qualitative approach, we used content analysis to identify themes from the feedback residents received from SPs and faculty observers. After themes were identified, we held a focus group with residents to determine which themes contained helpful feedback.

**Results:**

Residents found feedback from both SPs and faculty observers to be important, and how information was delivered was identified as the most impactful type of feedback. Residents appreciated positive feedback from SPs and faculty, especially when aimed at reinforcing a specific behavior that was performed well. Feedback that residents identified as particularly helpful, although not commonly provided, was the recognition of a potential unconscious bias in an encounter. Feedback the residents found less useful was discussing the medical specifics of the case. The most frequently provided types of feedback were not necessarily the most helpful feedback for both SP and faculty feedback.

**Discussion:**

OSCEs can be an effective tool to provide child neurology residents with immediate feedback on their communication skills with difficult conversations in a safe environment. Residents find value in feedback from both the SPs and faculty observers. Certain elements of feedback are more helpful than others. This knowledge could be used to develop an assessment tool to guide feedback from child neurology-specific communication simulation.

## Introduction

Child neurologists are tasked with unique challenges in communication. To name just a few, they need to be able to effectively explain complicated neurologic concepts, appropriately address patient and family concerns in situations with limited information, and compassionately provide emotional and mental support during hard conversations. These difficulties are rooted in the fact that child neurology has many complex disorders with a wide array of prognoses and treatments. Furthermore, child neurologists must often juggle the patient-parent-physician triad and adjust communication for various developmental levels.^1-3^ Studies have shown that how information is delivered to patients and families can significantly affect their ability to comprehend and cope with neurologic disorders.^4-9^ Given that these challenging conversations occur regularly in both inpatient and outpatient settings, child neurologists need to develop communication skills to adeptly navigate these situations early in their careers.

In 1999, the Accreditation Council of Graduate Medical Education classified interpersonal and communication skills as one of their core competencies.^10^ While communication skills do not necessarily improve with clinical experience alone, they can be taught and have been widely incorporated into medical school and residency curricula.^11,12^ Residencies have explored different methods to help develop these skills among their trainees intentionally.^13-16^ Published communication-centered curricula have been implemented for residents ranging from single-day workshops to longitudinally integrated programs using a variety of teaching methods.^17-20^ Active training techniques such as role-playing and feedback, as opposed to more passive techniques such as modeling or oral presentations, have proved to be more effective strategies in teaching communication skills.^21^ However, limited literature exists regarding effective communication training during child neurology residency.

The objective structured clinical examination (OSCE) has been a widely used assessment tool in medical education to help learners develop clinical and communication skills. The advantage of this tool lies in the ability to observe learners in real time within a controlled and standardized environment. Standardized patients (SPs), actors who portray patients, can be incorporated into the OSCE to act in ways that mimic realistic patient and family interactions. Direct and immediate feedback can be given from SPs and evaluators on the learner's performance. OSCEs have been incorporated into multiple adult neurology residency programs with data supporting their utility and reliability. Examples of OSCEs included evaluating neurology residents' ability to deliver difficult diagnoses such as amyotrophic lateral sclerosis and psychogenic nonepileptic seizures, acquiring tissue plasminogen activator consent in acute stroke management, and disclosing prognosis after hypoxic ischemic brain injury.^22-26^

We had previously developed a set of communication-focused OSCE cases that residents had found useful to their education and the overall feedback received was valuable.^27,28^ Our aim for this study was to expand on our previous work using qualitative analysis of the feedback provided to child neurology residents by SPs and faculty evaluators during a communication-focused OSCE, looking for common themes and to identify which elements of communication feedback were the most valuable to the residents.

## Methods

### Structure of the OSCE and Feedback

We previously developed and piloted 9 OSCE scenarios with challenging but common clinical cases in child neurology. We selected three of the scenarios (an adolescent with migraine headaches and their parent, a pair of parents of a neonate with severe hypoxic ischemic encephalopathy, and a parent of a school-aged child with staring spells) to use for this study. Child neurology residents of all postgraduate years (PGY-3 - PGY-6) participated in the OSCE. The program was also open to adult neurology residents (PGY-2 - PGY-4). The event took place at The Ohio State University College of Medicine's Clinical Skills Education and Assessment Center (CSEAC) with rooms akin to clinic examination rooms with full audiovisual recording capabilities. SPs were identified from a pool of trained and experienced SPs in the college's program.

Each OSCE event contained 3 clinical case encounters with SPs portraying parents of a pediatric patient or an adolescent patient. Each case began with a clinical vignette describing the scenario. Instructions included counseling or discussion objectives to complete during the timed encounter with the SPs. The case materials and door instructions for each case have previously been published.^27,28^ The information on the door instructions was sufficient for the resident to start the encounter from the point of education and counseling without requiring time for complete history taking and examination. All residents rotated through 3 40-minute stations for each of the clinic case encounters. This included a 20-minute encounter followed by a 10-minute feedback session with the SPs and then another 10-minute feedback session with a faculty observer.

SPs received training beforehand about the clinical cases and skill level appropriate for the resident participants. Each of the examination rooms had a 1-way mirror allowing a faculty member to watch and listen using headphones to each of the clinical encounters. The faculty member did not listen to the feedback session between the resident and SPs to not influence the feedback given by the faculty member. Faculty raters underwent training to ensure reliability and consistency across the various clinical cases. Faculty observers were instructed to provide feedback to the residents on how they conveyed information in the encounters, with the focus on communication, not on the medical specifics of the case.

### OSCE Feedback Transcription and Analysis

Audio and video were recorded for each of the 40-minute stations using in-house software at the CSEAC. Text transcription was manually generated for both SPs and faculty feedback sessions for each of the 3 cases for all participants by watching the recordings. Transcripts were deidentified with speakers labeled resident, SP1, SP2, or faculty. Qualitative analysis was performed on each of the transcripts to identify themes.

SP and faculty feedback were analyzed separately. Transcripts were reviewed by one of the authors to identify themes in the feedback, and a coding document was generated. Thematic analysis was grounded in the research question of what makes helpful constructive feedback. Phrases relevant to the theme were copied into the coding document, and some phrases were representative of multiple themes. Transcripts were reviewed in detail until themes reached saturation, subsequent transcripts were skimmed for new themes not previously found. An audit trail was kept throughout the process, notating initial observations. Peer debriefing was used if there was uncertainty about a theme. Member checking was performed by two other authors (M.A.R. and P.W.) to ensure the themes were coded appropriately.

### Focus Groups and Analysis

A focus group of residents was held with the participants following qualitative analysis of the feedback session transcripts to discuss identified themes. Focus groups were led by a facilitator to discuss the compiled list of the themes identified during SP and faculty feedback sessions. Each resident was encouraged to share their thoughts on the importance and relevance of the themes. The focus group was held on Zoom and audio recorded which allowed for an automatically generated transcription of the session. Residents were deidentified and referred to by a chosen pseudonym during the focus groups. Member checking was also performed with the residents to identify if any themes were missing. The generated transcription was reviewed to gather feedback from residents regarding the identified themes and any other thoughts they had regarding the OSCE exercise. OSCE Focus Group Interview Protocol is included in supplemental materials.

### Standard Protocol Approvals, Registrations, and Consents

The study was reviewed and approved by Nationwide Children's Hospital Institutional Review Board. Residents who participated in the focus group provided written consent to participate.

### Data Availability

Anonymized data not published within this article will be made available by request from any qualified investigator.

## Results

### Objective Structured Clinical Examination

Fourteen child neurology residents participated in the communication-focused OSCE in November 2021. All 3 cases were completed by each resident, and all OSCEs for this study were completed on the same day. This resulted in a total of 42 faculty feedback recordings (420 minutes) and 42 SP feedback recordings (420 minutes).

### Content Analysis

After manual review of transcriptions of faculty and SP feedback recordings, common themes were identified from the qualitative analysis. Sixteen themes were identified from the SP feedback sessions in supplemental materials eTable 1, and 14 themes were identified from the faculty feedback sessions supplemental materials eTable 2. Overall, the themes spanned a wide range of communication topics. Ten of the themes were shared by both SP and faculty feedback: encouraging self-reflection, demonstrating caring, information delivery, checking for understanding, case-specific advice, body language/nonverbal communication, use of jargon, rapport building, directly engaging the adolescent patient, and kudos/nice comments. The themes unique to the feedback from the faculty were information gathering, use of silence, control of the conversation, and use of filler words. The themes unique to the feedback from the SP were allowing the resident to ask questions to the SP(s), reaching an agreement, recognition of potential unconscious bias, physical touch, spending time with the patient/family, and repeating words (Figure). In both SP and faculty themes, case-specific advice was the most common feedback given. Information delivery, encouraging self-reflection, and directly engaging the adolescent patient were other commonly used feedback themes that were given by both SPs and faculty.

**Figure:**
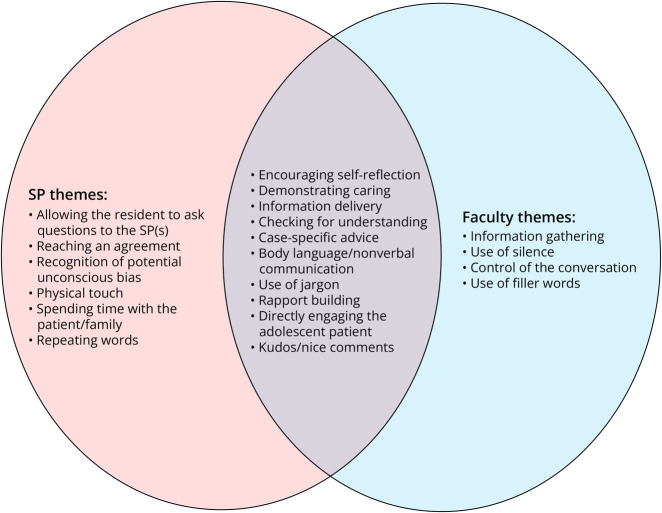
Distinct and Overlapping Faculty and SP Themes SP = standardized patient.

### Focus Group

Five residents who participated in the OSCE agreed to participate in a focus group. All the residents reported finding the experience helpful. Common words residents used to describe the experience were *feedback*, *tearful patients*, *hard or tough conversations*, and *nervous or anxious*. One resident reported some skepticism before the experience about how true to life the cases would be. All the residents also agreed that this OSCE affected their future practice. For the adolescent with the migraine scenario, one resident noted that the “adolescent” SP gave feedback that they liked when the resident asked the parent SP to step out of the room, and “that is something that they appreciate me [the resident] doing and it's something I continue to be intentional about, even though I think in neurology we often don't do that.” Another resident expressed how in the context of delivering bad news that “the change in my practice was to lengthen that space, that silence, a little bit more and just allow the information to simmer.”

Four residents recognized that the most helpful feedback they received was from the SPs, and 1 resident recalled their most useful feedback from a faculty observer. The most helpful SP feedback themes identified were engaging with the adolescent patient, use of jargon, information delivery, kudos/nice comments, body language/nonverbal communication, and recognition of potential unconscious bias. In particular with information delivery, residents revealed that this kind of feedback emphasized the need to deliver information differently depending on the patient, checking for understanding, and asking permission to proceed with delivering information. Recognition of unconscious bias was particularly insightful for residents in cases where there may have been an unconscious bias the resident was unaware of. In some cases, the residents received feedback that they were exclusively talking to the “mom” in the scenario and ignoring the “dad” and similarly, spending more time talking to the “parent” than the “adolescent” patient. Residents noted that feedback regarding behaviors that are highly variable among individuals such as physical touch was not as helpful.

The most helpful faculty themes were information delivery, use of filler words, kudos/nice comments, use of jargon, control of the conversation, use of silence, and body language/nonverbal communication. All of the residents especially appreciated the positive feedback that helped reinforce behaviors that were performed well and should be continued. Residents also pointed out that case-specific advice such as medical details is a less helpful type of feedback in this communication-focused activity. One resident also noted that feedback regarding rapport building is welcomed while taking into account the artificial nature of the situation.

## Discussion

Our aim for this qualitative study was to better understand the types of feedback that residents find to be most valuable during communication-focused OSCEs. Our study offered a unique opportunity to analyze feedback sessions from both SPs and faculty from multiple different scenarios as well as receive direct feedback from the residents who participated in the OSCEs. We were able to identify specific areas of feedback that child neurology residents found more or less helpful for communication-focused OSCEs. Overall, residents want feedback on how information was delivered, not what information was delivered. With information delivery, residents found it helpful to know if they spoke in a manner that was easy to follow vs circuitous or confusing to patients. Similarly, faculty feedback regarding medical knowledge specifics such as medication side effects was less useful for residents in this setting.

Most residents felt feedback from the SPs was the most valuable; however, one also identified the value of faculty feedback, suggesting that both perspectives are important. Residents rarely get the opportunity to elicit direct feedback from patient and/or family perspectives in real-life clinical practice. OSCEs can serve as a unique opportunity to hear feedback immediately following such encounters even if they are artificial environments. *Recognition of unconscious bias* was a particularly helpful theme unique to the SP feedback. An explanation for this could be that this type of feedback is more easily identified by an individual participating in the scenario as opposed to a third-party member who is observing the encounter. For example, the “dad” and “adolescent” SPs were able to identify encounters where they felt less attention was paid to them by the residents. Other studies have found value in the SP feedback, and learners can appreciate this feedback just as much or more positively than physician feedback.^29-31^ On the other hand, control of the conversation was a theme identified only from faculty feedback. As an observer, the faculty had a more global view of the encounter and could identify if a particular individual dominated the conversation or if the resident did not give the SP opportunities to talk. The faculty could then give feedback based on their own experiences from similar situations as to how to improve.

Given the differing backgrounds, experiences, and expertise of SPs and faculty, both parties are uniquely positioned to focus on different aspects of feedback. While there were themes distinct to SP or faculty feedback, a majority of the themes were shared by both. This suggests that there is probably a large overlap in the overall types of feedback that both groups can provide. Common themes can serve to further reinforce a point of feedback, and both parties can also give different perspectives and nuances. Furthermore, child neurology residents at all levels of training participated in the OSCE and were invited to participate in the focus group; therefore, feedback was not segregated based on level of training. Future studies could evaluate potential differences between different levels of training.

Of interest, the most frequently given feedback themes were not necessarily the most helpful feedback based on resident responses from the focus group. While case-specific advice was the most common type of feedback given, residents believed this feedback was not as helpful given their desire to assess their communication skills and not the associated medical knowledge used in the OSCE case. Helpful faculty themes such as control of the conversation, use of filler words, use of silence, and use of jargon were only given a handful of times. Similarly, recognition of unconscious bias and use of jargon were helpful SP feedback that was rarely given to residents. This discrepancy in helpful feedback vs commonly given feedback can be addressed in the future by priming both faculty and SPs with the types of feedback residents find most valuable before the start of the OSCE. This could be performed through rater training or the development of an assessment tool or checklist. By knowing which types of feedback are most and least helpful to residents, faculty and SP can better provide more specific, actionable, and personalized feedback for each learner.

The feedback themes that we identified in this study, when compared with other existing communication conceptual frameworks, reveal many similarities but also some distinct differences. Our institution had used the Gap-Kalamazoo communication skills assessment form in previous years with these same OSCE cases.^32^ This widely tested and used assessment tool includes 9 categories: Builds a Relationship, Opens the Discussion, Gathers Information, Understands the Patient's and Family's Perspective, Shares Information, Reaches Agreement, Provides Closure, Demonstrates Empathy, and Communicating Accurate Information. Our identified themes cover all of these same themes except Opens the Discussion.^32,33^ While many of the residents did appropriately elicit patient/family concerns and allowed space for them to complete their opening statements, interestingly, this aspect of the encounter was not commonly discussed during the faculty or SP feedback sessions. Our study had several themes that focused on how communication was delivered such as body language/nonverbal communication, physical touch, use of filler words, recognition of potential unconscious bias, and use of silence which are not explicitly emphasized in the Gap-Kalamazoo communication skills assessment form. Several of these themes were also some of the most helpful for residents.

Given that the chosen OSCE cases were purposefully emotionally charged and required residents to give difficult diagnoses, the nuances of how information is conveyed become even more important to help patients/families during difficult conversations. While the Gap-Kalamazoo assessment form can serve as an initial outline to help guide communication feedback, this instrument is very broad and there may be aspects that are missing for different specialties or specific situations. One study with internal medicine residents identified new feedback themes of patient education, communication intelligibility, and thoroughness that expanded on existing communication frameworks.^34^ Similarly, this suggests that there may be specific communication skills that are emphasized and necessary in child neurology that are not adequately assessed within the Gap-Kalamazoo communication skills assessment form. Our study ultimately allowed us to better understand how to improve communication training for child neurology residents. Future studies are needed to validate these identified feedback themes to evaluate their utility in assessment forms and how they would translate into real patient encounters.

The results of our study should be interpreted within the context of its limitations. The extended length of time of 21 months between the OSCE and the subsequent focus group likely affected the ability of the residents to recall specifics from their feedback sessions. As a result, there may have been themes and discussion points that could have been missed or forgotten by residents after such a long period. Furthermore, not all residents were able to participate in the focus group creating the potential for sample bias. The size of the pool of residents may have led to an underrepresentation of the breadth of feedback regarding the identified themes and may limit the generalizability of our results.

While residents can provide helpful insight into the OSCE experience as direct participants, there is also a limitation to the learners' ability to indicate what is helpful and unhelpful feedback. Residents may not have the insight to know which points of feedback are most important for future practice given their level of training. Thus, taking into account that resident feedback provides one perspective in evaluating this OSCE experience, we could look into other perspectives, such as faculty and SP feedback, as another method in evaluating types of feedback.

Our study only used 3 of 9 OSCEs scenarios within the previously created set given available resources. Given that these cases have been previously published in the literature, it is possible the child neurology residents obtained the cases before the OSCE, and that prior knowledge may have affected their performance on the assessment. While there are overarching communication themes and skills that are applicable in many situations, each of the cases focused on a unique situation that may elicit case-specific communication skills. Thus, the remaining 6 scenarios may have elicited other kinds of communication feedback from SPs and faculty that the residents could have identified as helpful. However, we understand that it is impossible to capture the entire breadth of child neurology even when expanding the number of different cases. Further research can expand on the breadth of communication feedback and themes to further help develop child neurology resident communication skills. This knowledge could be used to develop an assessment tool to guide feedback for child neurology-specific communication simulations.

## Glossary

CSEAC: Clinical Skills Education and Assessment Center
OSCE: objective structured clinical examination
SP: standardized patient
